# Central venous catheterization: the cephalic vein access

**DOI:** 10.1186/s13054-022-04031-y

**Published:** 2022-06-08

**Authors:** Shouyin Jiang, Yehua Shen, Xiaogang Zhao

**Affiliations:** 1 Department of Emergency Medicine, The Second Affiliated Hospital, Zhejiang University School of Medicine, Hangzhou, 310009 China; 2 Key Laboratory of the Diagnosis and Treatment of Severe Trauma and Burn of Zhejiang Province, Hangzhou, China; 3 Zhejiang Province Clinical Research Center for Emergency and Critical Care Medicine, Hangzhou, China; 4 Research Institute of Emergency Medicine, Zhejiang University, Hangzhou, 310009 China; 5 Department of Radiology, Children’s Hospital, Zhejiang University School of Medicine, Hangzhou, China; 6 National Clinical Research Center for Child Heath, Hangzhou, China

## Dear Editor,

Establishment and the choice of appropriate location of central venous line can be occasionally very difficult for critically ill patients [1]. Body weight, occurrence of clavicular fracture, infection risk and so on are determinant factors with regard to the location choice of cannulation [2, 3].

**Fig. 1:**
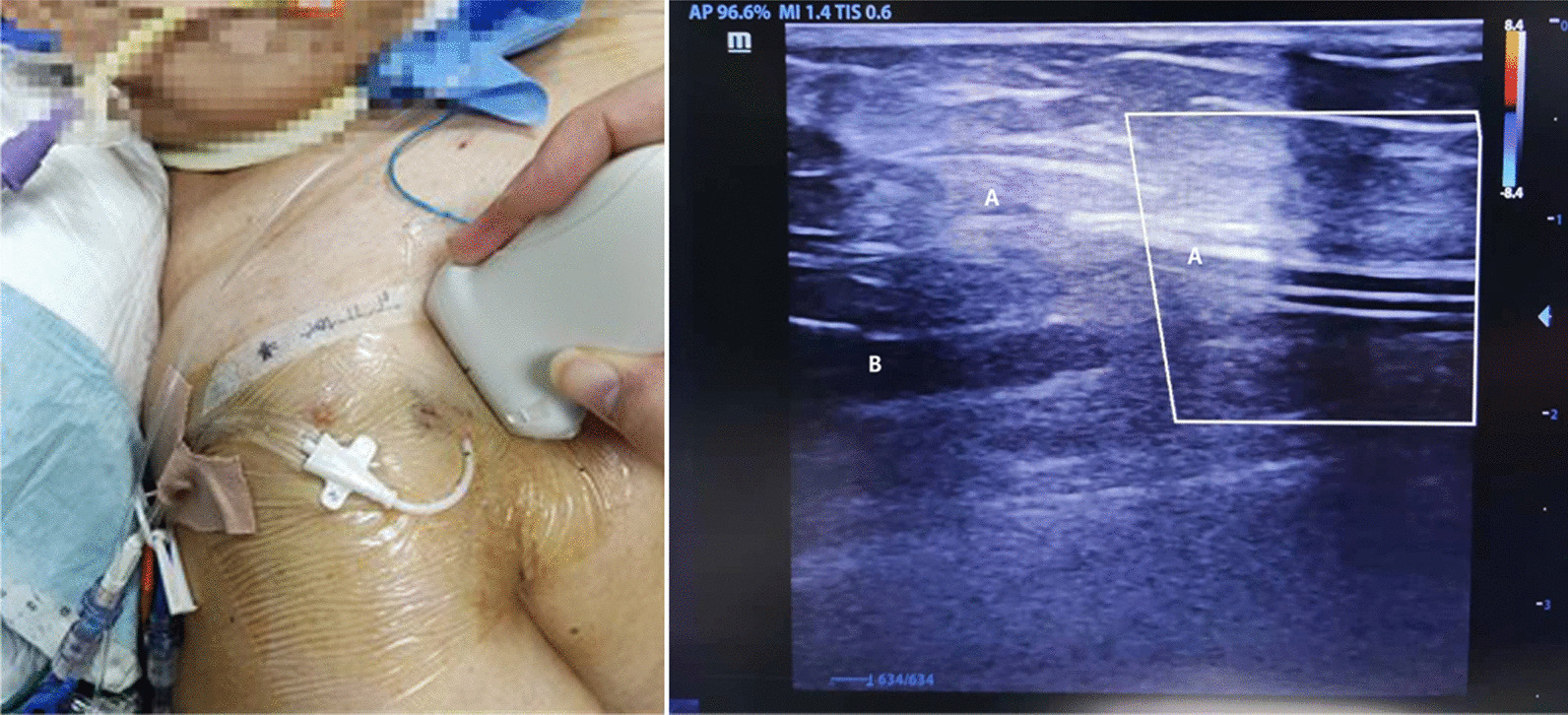
Puncture site for the cephalic vein access (left). Double track sign represents the central venous line (right). A, cephalic vein; B, axillary vein

A severely injured 81-year-old obese woman (body mass index of 28) was presented to the Emergency Intensive Care Unit (EICU) due to multiple organ failure (neural, respiratory, cardiac, circulatory, renal). She developed severe acute respiratory distress syndrome (ARDS) quickly after injury insult. Due to persistent acute renal insufficiency during EICU stay, she also received continuous renal replacement therapy (CRRT) through percutaneous catheterization of the left internal jugular vein. Thirteen days after admission, she required to change the right supraclavicular central venous line due to continuous vasopressor support and invasive hemodynamic management for heart failure and ARDS. Because there was no other appropriate route, we decided to insert the cannulation through the right cephalic vein, which was measured at 0.28 cm in diameter under ultrasound. The cannulation was successfully performed guided by ultrasound through the Seldinger technique, with the inserted length of 20 cm suitable for monitoring of central venous pressure (Fig. 1). Daily bedside ultrasound did not detect thrombosis within the cephalic vein.

There has been no case report in the literature introducing percutaneous catheterization of central venous catheter through cephalic vein access. However, the cephalic vein access has been used extensively for cardiac implantable electronic device placement through venesection with acceptable complication rates [4, 5]. The cannulation operation under guided by ultrasound can be easier in increased intrathoracic pressure due to venous filling. Our case demonstrated that the cephalic vein approach for central venous catheterization is safe without injury to artery, thorax, and nerve, which should be considered in selected critical care patients (e.g., cannulation difficult due to morbid obesity with no proper puncture site while still require monitoring the central venous pressure).

## Data Availability

The datasets used and/or analyzed during the current study are available from the corresponding author on reasonable request.
